# Supplementary material for: EPANet-KD: Efficient progressive attention network for fine-grained provincial village classification via knowledge distillation
Source: PLoS One. 2024 Feb 15;19(2):e0298452. doi: 10.1371/journal.pone.0298452 (PMC10868776; doi:10.1371/journal.pone.0298452)
Supplement: Attachment — Submitted filename: Response to Reviewers.docx [file pone.0298452.s001.docx]

1. Is the manuscript technically sound, and do the data support the conclusions?

The paper validates the effectiveness of the proposed method via experimentally quantitative and qualitative comparison with state-of-the-art classification methods. Meanwhile, ablation experiments are conducted on the proposed PAM and SAD to further validate the effectiveness of the proposed method. Finally, further intuitive visualization is explained with attention graphs and t-SNE.

3. Have the authors made all data underlying the findings in their manuscript fully available?

All datasets and code in the paper are publicly available from <https://github.com/Jack13026212687/EPANet-KD>

Reviewer #1: In this manuscript, the authors constructed a new dataset of the first Provincial village and proposed a PAM module to focus progressively on salient objects in the channel and spatial dimensions, and a SAL distillation strategy to reduce the number of parameters and computational effort. In addition, the authors compared EFPCNet-KD network with the state-of-the-art 7 methods to obtain a more advanced performance. There are some issues that need to be addressed:
1.In Methods, does the PVCNet-KD network refer to the EFPCNet-KD? The authors should clarify the differences between these two methods.

Both PVCNet-KD and EFPCNet-KD refer to EPANet-KD and have been harmonized throughout the text to all correct to EPANet-KD.

2.This paper is not well written. There are many mistakes in spelling, punctuation, and incomplete sentences in the manuscript.

We do feel sorry for the trouble. The spelling errors, punctuation and incomplete sentences have all been corrected in the manuscript.

3.The figures provided in the manuscript are not clear.

Thanks for your comment. All diagrams in the manuscript have been corrected to clear diagrams as suggested.

4.The size of font in the tables is inconsistent. The authors should revise the format of the tables.

Font sizes and formatting in all tables in the manuscript have been correspondingly corrected in all cases.

Reviewer #2: This article mentions the challenges of traditional village classification and identification, and an efficient attention-based fine-grained traditional village classification network (EFPCNet) is designed. This paper proposes a new knowledge distillation method with balanced probability distribution,Specifically, the probability distribution balancing loss MAE is introduced, which balances the probability distribution of the classification output of the softened EFPCNet-teacher network with the classification output of the EFPCNet-student network, as a way to transfer teacher knowledge to the student network. The results show that the method proposed in this paper achieves the best performance on the test data set. However, I think the method innovation in this article is limited, and there are some flaws and deficiencies in the method description and experiments. The specific problems are as follows.

Some suggestions are as follows:
1. The introduction discusses the importance of village research from a humanistic perspective. It does not involve relevant research on traditional villages using machine learning or deep learning methods. It does not technically emphasize the research questions and research difficulties of this article.

References [8-12] in the introduction section of the newly revised paper are supplemented with relevant research on traditional villages using deep learning methods. In addition, the ending sentence of each paragraph in the introduction section technically emphasizes the research problem and research difficulties of this paper.

2. The proposed method part lacks innovation, and the description of the method part is too brief. It is more of a simple combination of existing technologies. The author did not emphasize the technological innovation and improvement part of this article.

The paper constructs the first traditional village fine-grained categorization dataset PVCD. In addition, two innovations, namely PAM and SAD, are proposed. The newly revised paper provides a detailed introduction to PAM and SAD. Both the introduction and the methods section of the paper emphasize the technical innovation and improvement part of the paper.

3. Please add how to calculate the MAE in Formula 6 and the SAL and CEL losses in Formula 7.

Equation 4-9 of the newly revised paper unfolds how MAE, SAL, and CEL are calculated.

4. The author should give some sample pictures of the data set constructed in this article.

Thanks for your comment. A sample of the constructed traditional village fine-grained categorization dataset PVCD is given in the newly added Figure 2.

5. The quality of all pictures in this article seriously affects the understanding of the paper work, and the author should provide higher quality illustrations.

All figures in the full text of the new revised version have been revised to high quality.

6. The paper lacks specific explanations of some symbols in the formula, such as XxSigmoid in Formula 1 and xSigmoid in Formula 3.

Part B of the Overview in the Materials and methods section explains that it is sigmoid in PAM.

7. There are some writing errors in the paper, please check carefully, such as: igh, ablation.

Writing errors in the newly revised paper have been corrected throughout.

8. The experimental part is imperfect and lacks visual analysis of village classification.

The newly revised paper further intuitively visualizes and explains the fine-grained classification of traditional villages through attention graphs and t-SNE.
