# Supplementary material for: EPANet-KD: Efficient progressive attention network for fine-grained provincial village classification via knowledge distillation
Source: PLoS One. 2024 Feb 15;19(2):e0298452. doi: 10.1371/journal.pone.0298452 (PMC10868776; doi:10.1371/journal.pone.0298452)
Supplement: Attachment — Submitted filename: Response to Reviewers.docx [file pone.0298452.s002.docx]

Reviewer #1: After reviewing the paper again, it seems that it has improved slightly. However, there are still some minor problems:

1.In the paragraph explaining the formula symbols in the paper, the symbols in the formula are inconsistent with the size of font in the main text.

The consistency of the formula symbol sizes in the paper and the formula symbol sizes in the formulas have all been revised.

2.The labels of the figures in the paper are inconsistent. Please check carefully.

The labeling in the figure in the paper and the label name consistency throughout the text have all been revised.

3.Is the font in the table too small? Please modify it according to the journal's requirements.

The font sizes of all tables in the paper have all been modified according to the journal requirements.

Reviewer #2: Some suggestions are as follows:

1. The author should explain why EPANet-Teacher and EPANet-Student use different backbones, one uses Segformer-b4 and the other uses Segformer-b0.

Inspired by [33], a very deep heavyweight backbone network is chosen for high accuracy performance, and a shallow lightweight backbone network is chosen for efficient inference performance.The above opinion responses have been reflected in part A of the methodology

2. This paper proposes a new attention module PAM, which should be compared with other existing attention modules to highlight the advantages and innovation of the method.

The quantitative and qualitative results of the proposed PAM and other extant attention methods have been presented in Part C of the Discussion section in the paper, further demonstrating the superiority of the proposed PAM.

3. The latest literature review and citations are lacking. It is recommended that the author refer to the following literature.
”A collaborative gated attention network for fine-grained visual classification”
”Multi-label image classification with multi-scale global-local semantic graph network””Mining graph-based dynamic relationships for object detection”

This paper has reviewed and cited the three most recent literatures mentioned in the second paragraph of the introduction section.
